# Crossroads of well-being and compliance: a qualitative cohort study of visitor restriction policy during the COVID-19 pandemic, the Netherlands, May 2020-December 2021

**DOI:** 10.1186/s12889-024-17665-0

**Published:** 2024-01-13

**Authors:** Fatima Arrahmani, Annerike Gorter, Janneke Elberse, Anne H. Buitenhuis, Gerjo Kok, Pita Spruijt

**Affiliations:** 1 Corona Behavioural Unit, National Institute for Public Health and the Environment, Postbus 1, 3720 BA Bilthoven, the Netherlands; 2 Applied Psychology, Maastricht University, P.O. Box 616, 6200 MD Maastricht, the Netherlands

**Keywords:** Visiting restriction policy, Social distancing, Compliance, Well-being, COVID-19 pandemic, Cohort, Qualitative study

## Abstract

**Background:**

In this qualitative study we observed in-depth the impact of the visiting restriction policy (VRP, i.e. number of visitors allowed at home) on well-being and compliance during the COVID-19 pandemic to regulate infection rates.

**Methods:**

A cohort of 15 interviewees was followed throughout the COVID-19 pandemic in the Netherlands in 12 interview rounds (May 2020-December 2021). Every round semi-structured telephone interviews were conducted by a team of 8 researchers. In total 176 interviews were conducted.

**Results:**

This study showed that four categories can be identified when observing the impact of the VRP on well-being and compliance. For Resilient-Followers reasons for compliance were risk perception, following government rules, and for some having a small social circle. Because they accepted the situation, well-being was hardly affected. Resilient-Rulebreakers made their own risk assessment of people they met. Their well-being was hardly affected, because they experienced social rest and interpreted the measure in their own way. Suffering-Followers complied, because of risk perception, following government rules, and working in healthcare. However, the VRP had substantial impact on well-being, because social structures were disrupted. Suffering-Rulebreakers gave their own interpretation to the VRP, trying to find a balance between compliance and well-being. We observed that the categories were quite stable over time.

**Conclusions:**

The VRP appeared to be a measure with substantial impact on well-being for some, mostly because social structures were disrupted. The measure showed fluctuating compliance, in which feasibility and frequent changes in the VRP played a role. Well-being seemed related to the number of visitors that was allowed; a restriction of four visitors was feasible, while one visitor resulted in a negative breaking-point in resilience, which had an impact on compliance, even among the most compliant. Taken together, this study provides valuable insights into the implications of and compliance to a VRP during different phases of the COVID-19 pandemic, which may contribute to policymaking during future pandemics.

**Supplementary Information:**

The online version contains supplementary material available at 10.1186/s12889-024-17665-0.

## Introduction

During the COVID-19 pandemic the Dutch national government took measures to prevent and reduce the number of COVID-19 infections. These included social measures (e.g., distancing, visitor restrictions), hygiene measures and a test, trace and isolate strategy for those who showed COVID-19 symptoms. These measures have had a large impact on society. Many studies already examined the effects of such measures on well-being. For example, feelings of loneliness were higher during stricter measures related to social contacts [1–3]. Some studies showed marked fluctuations in mental health during different periods of the pandemic; i.e., a decline in mental health after stringency of measures increased and an improvement after measures relaxed [4–8]. People indicated this was partly because of missing friends and family due to the restrictions on social contacts.

**Fig. 1:**
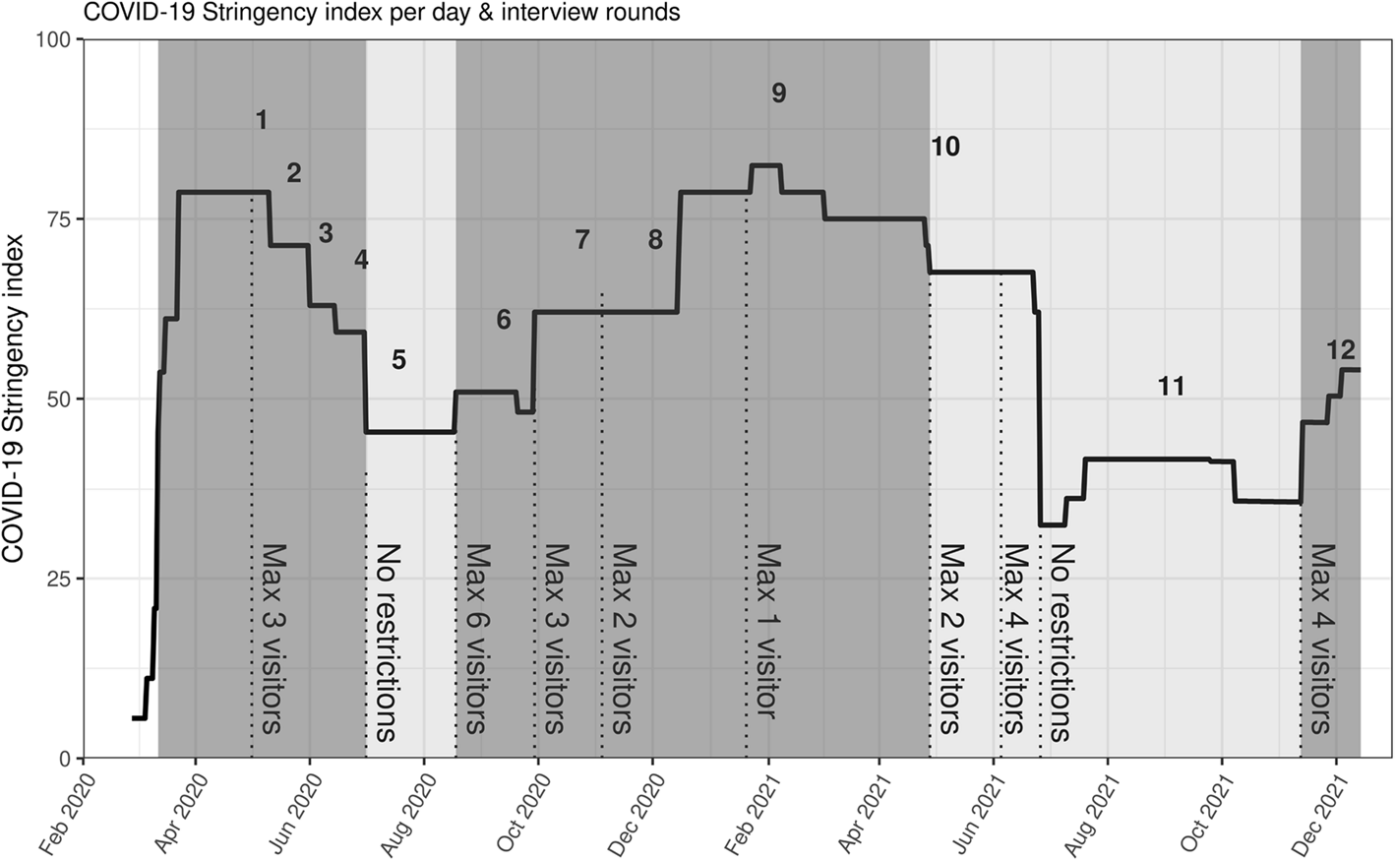
Visitor restriction policy per interview round plotted with COVID-19 stringency index. The COVID-19 Stringency index is calculated as a mean score of the stringency level of a government’s response to control COVID-19 infections on any given day, ranging from 0 (no measures) to 100 (strictest measures) [15]. The dark grey areas imply the period of restriction and the light grey areas imply the periods of relaxation of the COVID-19 restrictions

One of the preventive measures that impacted social life during the pandemic was the visitor restriction policy (hereafter referred to as VRP); i.e., governmental advice to limit the number of household visitors per day (see Fig. 1 and Appendix 1 for the VRP per period). Compared to other measures, VRP might be considered as a measure with great impact on daily life, as it hinders social contact which is important for well-being. Complying with social distancing measures, like VRP, requires a large effort, therefore, it is defined as a high-cost measure [9]. Several international studies reported on the adherence to these measures [10–13]. Mobility data showed that lockdown policies and vaccination impacted household visitation patterns. During the first lockdown a strong reduction in household visitations was observed. After relaxation of the VRP visitations increased again. Interestingly, during following lockdowns with reinforced VRP, there was a less strong decline in household visitation (i.e., lower adherence to the rules), which might be explained by multi-lockdown fatigue and inconsistent policy over time [11]. Other studies stated that compliance with social restriction rules (e.g., avoiding gatherings and going out, physical distancing) requires major behavior changes [11, 14].

Altogether, many studies have been conducted over the past two years regarding the general impact of COVID-19 measures on well-being. However, there is a lack of qualitative studies on individuals’ responses to changing COVID-19 measures over time and their well-being, specifically regarding VRP. Since VRP might be an impactful measure, it is relevant to examine further. The current study therefore aimed to examine how people experienced the VRP during different phases of the pandemic and how it affected their well-being. Qualitative studies provide insight into the underlying reasons and considerations people might have to cope with and comply with VRP and its impact on well-being. We examined how people cope with the VRP over a longer period and how it affects their well-being, by following a cohort of 15 interviewees for 21 months during the COVID-19 pandemic (May 2020 (start of the Corona Behavior Unit)-December 2021 (end of funding)) in 12 interview rounds.

## Methods

### Study design and recruitment

This study used an empirical and iterative research design. We conducted a research in an pandemic. Given the crisis situation and the rapidity of implementing the measures we had to be flexible and act on the situation with the information and capacity that was available.

For the first interview round (May 1st, 2020) 34 individuals were selected from participants of the COVID-19 questionnaire ‘research on behavioral measures and well-being’ [16] of the RIVM (National Institute for Public Health and the Environment), GGD GHOR Nederland (Association of Regional Public Health Services and Regional Medical Emergency Preparedness and Planning offices in the Netherlands)and 25 GGD’s (Municipal Health Service) [2]. In the first round of the questionnaire, ‘research on behavioral measures and well-being’, participants were asked if they were willing to participate in further in-depth research. Among the participants who agreed, we selected a sample of 36 participants, based on the capacity of the team of researchers. Of the 36 participants we eventually interviewed 34 participants. Two participants could not be reached. This selection was based on the participants’ age, education, urban or rural living environment, health situation and current living situation, of which a random sample was invited to participate in the interviews. These background factors were selection because the research thought those factors that could explain adherence and non-adherence to the measures taken by the government. During the first interview round, interviewees were asked if they were willing to participate in an interview cohort. They all agreed. For the cohort, we attempted to create an optimal distribution of age, gender, education, place of living (geographical distribution in the Netherlands and distribution of rural and urban areas), well-being and compliance with COVID-19 measures (Table 1). Therefore, 17 out of 34 participants were selected to participate in the cohort. Although the original number of interviewees was higher, due to the aims of this research and capacity of the research team, we focused only on the cohort interviewees (*n* = 17), since our goal was to examine the effects of changing the VRP over time on the behavior of the participants and how they coped with the changes. This study was conducted following the RIVM guidelines and regulations. In line with the Central Committee on Research Involving Human Subjects (see https://english.ccmo.nl/), the questionnaire study does not meet the requirement of the Law for Research Involving Human Subjects (WMO) and was exempted from formal ethical review. Informed consent was obtained from all interviewees included in the study. All interviews were recorded with the consent of the interviewee and participants gave consent for publishing their anonymized answers.

**Table 1:** Background information interview cohort (*n* = 15) (information based on information from all interview rounds)

*Interviewee nr*	*Year of birth*	*Educational level*	*Gender*	*Migration background*	*Living in rural or urban area*	*State of employment during interview series*	*Self-reported vulnerable health*	*Household composition*	*Vaccinated with at least one COVID-19 vaccine*^a^
*1*	1959	low	male	no	rural	unemployed/disabled	yes	lives with partner and two children	yes
*2*	1948	high	male	no	urban	retired	no	lives with partner	yes
*3*	1955	middle	male	yes	urban	employed, worked on location at an airport	yes	lives alone	yes
*4*	1954	high	female	no	urban	employed on location in healthcare	no	lives alone	no
*5*	1982	middle	female	no	urban	unemployed	no	lives with parents	yes
*6*	1995	high	female	no	urban	student: after studying she worked as a policy advisor	no	lives with partner	yes
*7*	1989	high	female	no	suburban	employed on location in healthcare	no	lives with partner and two children	yes
*8*	2002	low	male	no	urban	scholar: as a side-job works on location in a shop	no	lives with parents	yes
*9*	1978	high	female	no	urban	employed; works from home as teacher later as communication advisor	no	lives with partner	yes
*10*	1954	middle	male	no	urban	retired	yes	lives with partner	yes
*11*	1955	high	female	no	rural	unemployed	yes	lives alone	yes
*12*	1998	low	female	No	urban	student/ employed, worked on location in healthcare and teacher at school	no	lives alone	yes
*13*	1995	high	male	No	urban	Employed works on location in healthcare	no	lives with partner	yes
*14*	1995	high	female	No	rural	student/employed, works on location in social youth care	no	lives alone	no
*15*	1969	middle	male	No	urban	employed, works on location in healthcare	no	lives with partner and three children	yes

^a^The vaccination campaign started at 8 January 2021. We asked the vaccination status in the interview round 9 (11–14 January 2021) and in interview round 14 (24–31 August 2021)

Two interviewees dropped out during the interview period. One dropped out because of workload, the other did not give a reason. Therefore 15 interviewees were included in this study, who were interviewed at 12 time points.

### Data collection

Data was collected between 1 May 2020 and 1 December 2021. The intervals between the 12 interviews varied. At the start of the pandemic many measures were implemented by the government. During certain periods, communication from the government about COVID-19 and the frequency of changing the measurements decreased. The frequency of interviewing changes accordingly. During the 21-month research period there were phases of strengthening and relaxation of measures (see Fig. 1 and Appendix 1). As mentioned before, we used an iterative design, because we had to be flexible due to the crisis situation that was unpredictable. So we could keep up with developments in the Netherlands and deepen the relevant results of the previously mentioned quantitative questionnaire.

Every round a team of eight researchers conducted semi-structured telephone interviews over a five-day period. Interviews were recorded with the consent of the interviewee. Interviewers and interviewees were paired. Therefore, the interviewer was aware of the story of the interviewee and a relation based on trust was built between them.

In total 176 interviews were conducted. Four interviews were missed by interviewees, because of health problems, vacation or a lack of time due to a heavy workload. Interviews were transcribed verbatim. Anonymized data can be requested by the first author upon reasonable request.

### Instruments

Every round an interview guide was developed that was adapted and updated to the COVID-19 related situation at that time, to deepen the outcome of relevant results of the cohort questionnaire, to containing the most urgent and relevant themes for policy in the Netherlands. Before starting the interviews, the interview-guide was discussed with the interviewers. Since individuals were followed over time, we were able to monitor developments in well-being, reported behavior and thoughts regarding COVID-19 measures. Additionally, questions were asked about plans interviewees made for meeting their social contacts (e.g., for Christmas, birthdays), and their experiences, actual behavior and motivation afterwards. Interpretations of some interviewees’ responses about their compliance and well-being were verified by the interviewers during later interviews.

### Well-being

Every interview round interviewees were asked about their general well-being: i.e., how they were doing and whether there has been a change in their situation since the previous interview regarding their health, well-being, life, employment status and relationship(s). In different interview rounds questions were asked about well-being in relation to the measures at that time, the interviewees’ individual situation and the general situation of the pandemic.

### Visitor restriction policy

Questions about the VRP were specifically asked in round 2, 3, 6, 8, 9, 11 and 12. The VRP was only included in the interview guide if there was a change in the VRP rule. Interviewees were asked how they dealt with the VRP (excluding school, hospital, nursing home and all other assisted living medical facilities) and under what conditions they visited or received visitors at home. In other rounds, some interviewees mentioned the (impact of) VRP without specifically being asked about it.

Examples of questions about the VRP:

- Round 8: What measures are you thinking of in relation to the holidays?
- Round 9: How does the lockdown affects you (regarding social distancing and the VRP of 2 people)?
  Round 12: You may now invite a maximum of 4 visitors to your home. This number has been adjusted several times in the corona pandemic:
  - How do you handle this measure in practice? -Is this different from what you did before?
  - What is it like for you to move back along with the constantly changing number of people you may receive in your home?
- Round 12: Do you have any activities or events planned in the next few weeks where you expect the visitor measure to fray? If so, how will you handle them?

### Analysis

Interviews from round 1 to 9 were coded in MAXQDA by five research-assistants from the Radboud University under supervision of PS, JE and FA. They used a codebook developed by PS, JE and FA, based on the Health Belief Model, the COVID-19 measures, social dilemma theory and themes mentioned by the interviewees like well-being [16]. A conceptual framework about the VRP was not available at that time, as most studies found in literature date from the period after the interviews had started. During the encoding process the research-assistants discussed their findings with PS, JE and FA and coded each other’s interviews four times for interrater reliability.

The research-assistants were only available to code the transcripts of round 1 to 9. Because of lack of capacity round 10 to 12 were coded by focusing specifically on VRP (by researchers FA and AG). Next, all relevant codes in MAXQDA related to VRP and well-being were selected. Two researchers simultaneously checked if the selected fragments were indeed relevant and related to well-being, compliance and thoughts in relation to VRP.

The analysis was conducted using a thematic framework to structure high number of coded segments [17]. This framework showed several factors related to compliance and well-being, which will be described in the results. Both inductive and deductive methods were used. During analysis new themes were added to the framework. Both the storylines of each interviewee and the different phases of the pandemic were analyzed.

## Results

**Fig. 2:**
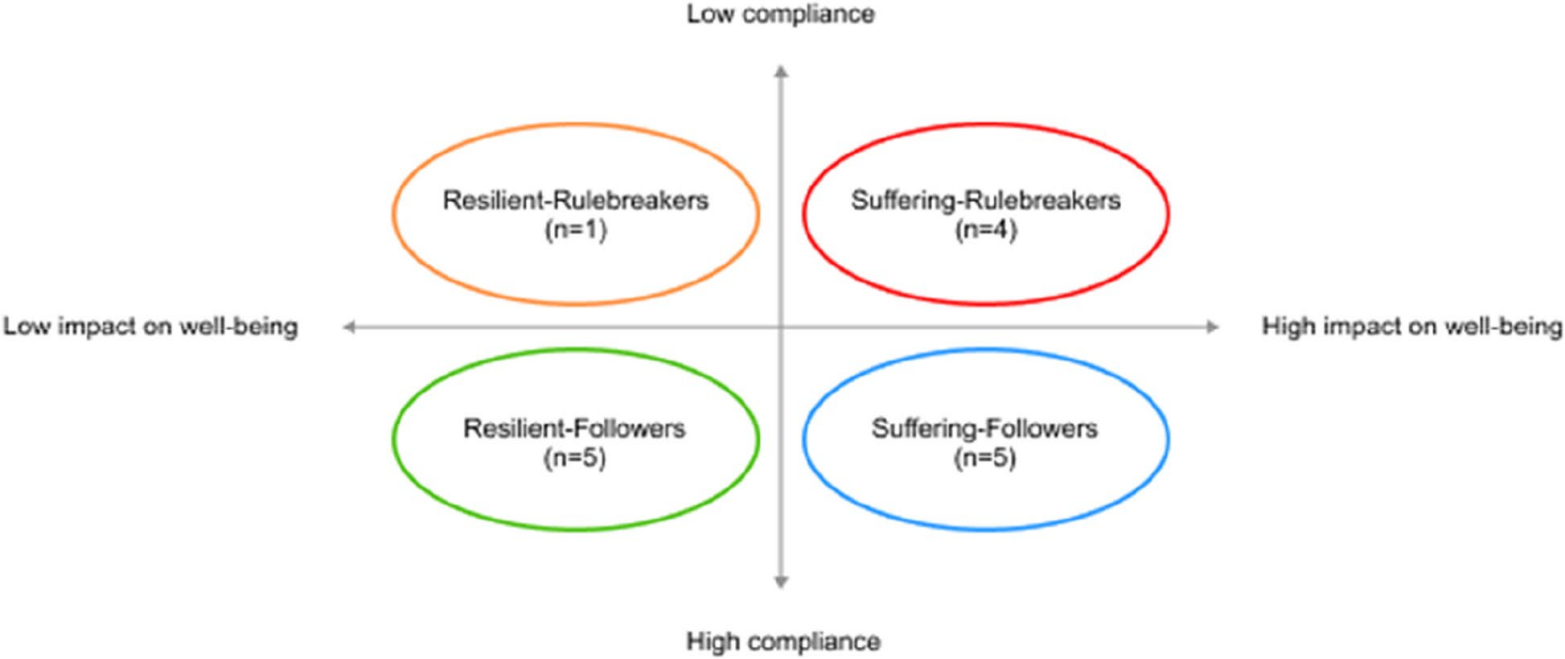
Graphic representation of four different categories in well-being and compliance related to VRP

When looking at the impact of the changing VRP on compliance and well-being of interviewees we observed that people dealt with it in very different ways as the impact on self-reported well-being also differed between interviewees. Some strictly adhered to the measure while others sometimes deviated from it, and some suffered mentally while others did not experience any problems. Results showed that the way in which interviewees dealt with VRP could be classified into four different categories. These four categories are based on a combination of interviewees’ reported level of impact on well-being (low to high) and level of compliance (low to high) (see Fig. 2).

These four categories were labeled as ‘Resilient-Followers’, ‘Resilient-Rulebreakers’, ‘Suffering-Followers’ and ‘Suffering-Rulebreakers’. ‘Resilient’ and ‘Suffering’ refer to the degree of impact of the VRP on well-being, ‘Followers’ and ‘Rulebreakers’ refer to the level of compliance to the VRP restrictions. It should be noted that these categories are not fixed, but gradual. Also the interviewees could deviate from the classified category in some rounds. Two researchers (FA and AG) independently assigned each interviewee to one of the categories, based on the initial interview, which showed full agreement. Additionally, the assigned category was verified with each interviewee in the last interview. All interviewees could relate to/agreed to their assigned category. For each category, we examined how interviewees dealt with the VRP during the pandemic. We analyzed factors that played a role in (non)compliance and looked at the impact on well-being. Different factors played a role in the four different categories (see Table 2), which are discussed in detail following the timeline of the pandemic below.

**Table 2:** Factors in compliance and well-being per category (information based on data from all interview rounds)

	**Factors for compliance**	**Factors for non-compliance**	**Factors for well-being**
**Resilient-Followers**	-risk perception -cue to action (government rules) -small social circle	-	-acceptance of situation -levelheaded
**Resilient-Rulebreakers**	-	-make own risk assessment -do what feels logical	-social rest -levelheaded
**Suffering-Followers**	-risk perception -cue to action (government rules) -working in healthcare	-	-large social network (disruption social structures -feeling sad, depressed or anxious
**Suffering-Rulebreakers**	-	-balancing between compliance and well-being -own risk assessment -do what feels logical -large social network (disruption social structures) -not willing to exclude someone -trust family/friends to not have the virus -social environment	-feeling sad, depressed or anxious

### Spring 2020 (May–June 2020) – increased stringency of VRP (three visitors) (*N* = 15)

For all categories different motivations for (non)compliance were observed. For Resilient-Followers, risk perception played a major role in compliance. Interviewees complied with the measure because they were afraid of a COVID-19 infection and/or belonged to a high-risk group (because of health problems or higher age). Even though they missed their contacts, they considered being careful not to get COVID-19 as more important.

*"On one hand you miss it, but you're also alert. If you're having face-to-face contact, it can also be risky, someone could infect you. So, it gives me mixed feelings. You miss it, but on the other hand, you know it's not beneficial either." – interviewee 5, female, 38 years old*

When it comes to compliance, several Resilient-Followers had a clear goal in mind: their own protection. In addition, interviewees mentioned that you 'just have to follow the government rules’. A few interviewees indicated that their social circle is small, so their existing social structures were not disrupted by VRP and, therefore, compliance required little to no adjustments. The well-being of Resilient-Followers was not (or hardly) affected by the measure, even though interviewees did miss having social contact. Acceptance of the situation seemed to play a role; Resilient-Followers seemed able to be resigned to the situation.

*"I do miss it, shaking hands and a hug, and other things too but yeah, it's just the way it is." – interviewee 5, female, 38 years old*

Resilient-Rulebreakers did not always comply with the measure. However, they believed they could reduce the risk by carefully making a risk assessment of the people they meet (e.g., only people with low-risk behavior). The measure had little impact on their well-being, partly because they did not strictly comply with the measure, but also because the measure provided social rest, despite missing social contacts.

*"I've sort of got used to it, but I do miss it a lot. But I don't think I'd want to go back to the old situation either, because I notice that I also really enjoy the weekends with the children and the peace and quiet." – interviewee 7, female, 30 years old*

Suffering-Followers did comply with the measure, but it had a substantial impact on well-being from the beginning of the pandemic.

*"The crisis doesn’t affect me, just the measures that are established. It feels like running into a wall, I feel anxious, because of sitting inside and the restrictions. It's not pleasant. If you're used to having lots of social contacts, eating out a lot, going to the gym, having an active life which is no longer possible, that doesn't really make you happy." – interviewee 10, male, 65 years old*

Several Suffering-Followers indicated they have many social contacts and were used to meeting in groups. Their social structures were disrupted by the measure and the adjustment they had to make was considerable. For example, one of the interviewees met with a large group of friends online, but digital contact compromised the quality of the contact:

*"When we normally meet with my group of friends, which is quite large, about fifteen friends, you easily talk in smaller groups and to different people from time to time. But online you can't really do that when you're all in a chatroom, because then you're all together the whole time, so that’s harder. I have the idea that the quality of conversations is much lower because you don't have the opportunity to have a real conversation. I do notice more and more that I miss that contact in person." – interviewee 6 female, 25 years old*

Despite the impact the measure had on well-being (such as feeling sad, depressed or anxious), Suffering-Followers still managed to stay compliant. They had several motives to comply, for example risk perception, wanting to follow government rules and working in healthcare.

*"We keep the social contacts at a low level, because we work in healthcare. We feel that we have a different responsibility to people, it would be different if we had worked for a regular organization." – interviewee 15, male, 50 years old*

For Suffering-Rulebreakers the measure also had a great impact on well-being, but unlike Suffering-Followers, Suffering-Rulebreakers did not always comply with the measure, for various reasons. First, they tried to find a balance between compliance and well-being.

*"The measures, of course, are always tough. Because you must find balance between trying to comply as much as possible and on the other hand keeping yourself happy enough, so to speak". – interviewee 8, male, 17 years old*

Second, interviewees considered it safe or ‘okay’ to invite more people if they were able to keep a distance and no one showed symptoms.

*"With friends I sometimes find it difficult. You tend to color outside the lines. So, I think that when the measure is four people maximum, and you invite five people and the fifth one can keep distance and has no symptoms, it is okay. Of course, you must pay attention to the maximum numbers, but if everyone is just healthy, what does one person more matter? Of course, you shouldn't invite ten or twenty more, but one more is not the end of the world." – female, interviewee 12, 23 years old*

Third, interviewees indicated that the rule did not fit someone’s social structure.

*"To me, it doesn't feel right to exclude friends because you're only allowed to invite three people, even though they belong to the group." – interviewee 14, female 25 years old*

Fourth, several Suffering-Rulebreakers indicated that, like Suffering-Followers, they had many social contacts and were used to meeting in groups. The VRP had such a large impact on their existing social structures and, therefore, their well-being, that Suffering-Rulebreakers deviated from the measure to meet their social needs. The analysis showed that interviewees felt better as a result.

*"Because I feel relaxed when being with friends and family. You could say ‘see your contacts digitally’, but for me that doesn't have the same value as face-to-face contact. So that's my excuse to see more people than allowed." – interviewee 14, female 25 years old*

Fifth, Suffering-Rulebreakers trusted family and friends would not have the virus.

*"It was an afternoon that we wanted to play cards with friends, and actually we were with more people than the measure prescribed. But we knew our friends were also no risk seekers and compliant, so we discussed it with them." – interviewee 9, female 41 years old*

Lastly, the people in the social environment of Suffering-Rulebreakers usually did not comply to the VRP, which allowed people to deviate from the measure together.

It should be noted that Rulebreakers in this cohort did try to comply with the measure at least a little. A high-risk perception was a reason to not completely disregard the measure. For example, interviewees worked in healthcare or had vulnerable people in their social environment. Instead of following the measure as prescribed, interviewees made their own well-considered risk assessment and interpreted the measure in their own way, to safeguard their well-being or because a rule did not make sense to them. In that way, they could justify their non-compliance, as for them, it felt like the right or logical thing to do.

### Summer 2020 (June – September 2020) *– *relaxation of VRP (no restrictions) (*N* = 15)

When the measure was relaxed, especially the Suffering groups experienced mental relief. They immediately felt better and frequently met with friends again.

*"It's going fine. The measures have been relaxed, so because of that my mood has brightened up a bit too. The measures had a great impact on social life, but now we have more social contacts, so relaxation of the measures has a positive effect." – interviewee 10, male 65 years old*

Although Resilient-Followers did not feel negative towards relaxation of the measure, they remained cautious about seeing others. Some interviewees remained stricter than the measure prescribed.

*"There are some family gatherings that I don't go to, because I read on the internet, and also on teletext, that most infections happen during family gatherings. So, I try to avoid that." – interviewee 5, female 38 years old*

This also applied to a few Suffering-Followers, who remained cautious because of their work.

*"I'll be very honest, if I didn't work in healthcare, I wouldn't be so concerned about the risk of infecting someone else in my work and wouldn't be so aware of the consequences. Now I feel responsibility towards other people because I’m a nurse. I don't want to risk infecting others. I've seen what COVID-19 does to you in hospital, how people get sick from it, die from it. So, I feel a very big responsibility to at least make sure I can't become one of the sources of infection." – interviewee 15, male 50 years old*

### Fall/ winter (October 2020-January 2021) – increased stringency of VRP (from max. six to three, two, one visitor) (October – November 2020 *N* = 15 and January 2021 *N* = 13)

When the VRP was reinforced as infection rates rose, Resilient-Followers complied with the measure again without difficulty. They accepted the new situation and adapted easily. Appointments that no longer fit the current measure were cancelled. For some interviewees compliance was easy, because they had a small social circle.

*"We don't make a big deal about that. It's just the way it is. I always assume it's temporary, that's in the back of your mind, so we should be able to do so." – interviewee 3, male 64 years old*

For Resilient-Rulebreakers, reinforcement of the measure did not have much impact on well-being either. Regarding compliance they indicated they were particularly selective about the people they chose to meet. However, the Suffering groups, who were frequently making appointments with groups of friends when there were no restrictions, faced severe challenges when the number of visitors was limited again. As the measure lasted longer and the number of visitors was limited further, it became increasingly difficult. For example, one of the younger Suffering-Followers indicated:

*"When I think about how much I'm at home compared to earlier, the difference is more than 50%. Before COVID-19, I was really doing much, visiting people and eating together. Every time you're really limited in what you can do. I think that's the whole thing, every time new measures are taken, you must change your plans again. And every time it just sucks a little bit more." – interviewee 6, female 25 years old*

These interviewees especially struggled with the rule because the maximum number of visitors did not match the number of friends they usually met. Suffering-Followers complied with the rule: they met others in smaller groups or sought creative solutions such as meeting online. Suffering-Rulebreakers, however, deviated from the rule when it disrupted their usual social structure. For several interviewees it did not feel right to exclude people who belonged to the same group. One Suffering-Rulebreaker said:

*"Of course, it feels kind of bad if you have a group of eight friends, like me, and not invite that last person. So, you’re allowed to invite six, but when I'm the seventh one, and another friend the eighth one, we may not come, so that’s really kind of bad.” – interviewee 8, male 17 years old*

He chose to deviate from the rule because not seeing his group of friends had more impact on him than getting COVID-19.

*"I think sometimes something, in this case a measure, has more impact than it should have. That would have had a bigger impact on me than getting COVID-19. The chance of getting seriously ill is small, I mean, still it could kill me. But you know, I could also die from riding my bike, which is also a risk I take daily. So, choosing to see my friends is just another risk I take daily. And for me, the risk of getting infected is worth it." – interviewee 8, male 17 years old*

Another interviewee mentioned that she chose ‘customization’ of the measure and weighed the risk:

*"I try to adhere to everything, but I also look at it as a kind of customization. If I think that five people in my house is also fine, we can keep enough distance and it does not give an extra risk, then I choose to do it, while it is not actually allowed." – interviewee 14, female 25 years old*

### Christmas and birthdays

During Christmas 2020, the VRP was relaxed and allowed three visitors (instead of two). The measure had a great impact on interviewees’ usual traditions and the setting in which one normally celebrated Christmas. In practice, interviewees dealt with the measure differently. Followers, for example, adjusted their Christmas celebrations to the maximum number of visitors that was allowed, celebrated it in small groups spread over several days or celebrated it online.

*"My uncle and aunt are going to make a Christmas dinner, so everyone picks up their meal and we're going to eat together behind the laptop. So, everybody eats Christmas dinner in their own house with their own household, behind the laptop. This is how our family can still celebrate Christmas together." – interviewee 6, female 25 years old*

Resilient-Rulebreakers indicated they did not strictly adhere to the allowed number of visitors. They reasoned that the spreading of visitors had the same risk as seeing each other at the same time. In terms of risk, three people were considered the same as three households.

*"My parents say, if we see one couple one day, and see the other couple the other day, the risk of getting COVID-19 is the same when meeting all together at the same time. Of course, you’re allowed to invite three people at Christmas. I wonder if that's three people, or if you can also interpret that as three households. After all, if we visit my parents as children, you might as well bring your partner, which doesn't really matter anymore if you live together.” – interviewee 7, female 30 years old*

One Suffering-Rulebreaker indicated she would not strictly adhere to the number if it would mean excluding family members:

*“You can invite a maximum of three people, but when there's four of us, then I'm not going to say at Christmas, 'hey sister-in-law, you can't come because you're the fourth person.' Then I would invite her of course. But meeting with the whole family like we normally do, we wouldn’t do that. It would probably be in smaller groups.” – interviewee 14, female 25 years old*

Several interviewees thought about which household it would be best to celebrate Christmas; the largest house would facilitate distancing, which would justify a few more visitors.

Most Resilient-Followers looked at the restrictions with Christmas level-headed; Resilient-Rulebreakers had mixed feelings, but also experienced social rest; the Suffering groups thought about Christmas as ‘not so much fun’, ‘unhappy’, ‘tough’, ‘disappointing’ and ‘very unfortunate’. Several interviewees noted that you must make something of it yourself. Some also found it quieter and less running around.

Regarding birthdays, interviewees also dealt with the VRP in various ways. Some interviewees cancelled their birthdays, others had a 'split birthday', which meant the birthday was celebrated in time blocks. For example, one Suffering-Follower described:

*"We had planned my son’s birthday before the new measures came in. We just canceled it and we now have split it up into three days with three guests maximum each day." – interviewee 15, male 50 years old*

### Mental breaking-point: one visitor

A mental breaking-point for the Suffering groups was the moment when the VRP allowed only one visitor (20 January 2021–28 April 2021). Suffering-Followers continued to comply with the measure (because of risk perception, following the government rule and working in healthcare), but the mental price they had to pay was increasing. For example, one of the interviewees experienced deep disappointment and sadness when she had to adjust the plans she made for the umpteenth time:

*"The new restrictions make me feel a little less happy. And then I say it mildly. At the last press conference, when the minister announced the maximum number is reduced from two to one, I was really sad. Mainly because each time there are more restrictions. First there were six people, then three, then two, and now one. Every time I think about how I should deal with it, and how I can ensure I can still see friends or have social contacts within the measures. Every time I've just succeeded in planning things within the measures, the whole situation changes again, and new measures come into force. And then I must start all over again, inventing and planning things. It's always a lot of disappointment. When seeing the press conference, I had to cry very hard. Just because I thought, I can't keep going anymore.” – interviewee 6, female 25 years old*

Another interviewee mentioned the uncertainty of whether scheduled appointments could continue, caused psychological pressure:

*"I think that, more than anything else, that brings psychological pressure. Every time a press conference is coming up, you are nervous that appointments that you’ve made earlier must be cancelled. When the number was reduced from two to one visitor, you had to cancel appointments again, which were not possible anymore." – interviewee 13, male 25 years old*

Whereas with a maximum of two or three visitors it was still possible to have some social contacts, with only one visitor allowed, the limit seemed to have been reached among interviewees. For example, one interviewee said:

*"We had quite a few contacts that we always visited as a couple. That's just all quite complicated to get that sorted out. I find that very difficult, I feel like there’s nothing fun possible anymore. In the previous situation, in which the number was three or two, it was often possible to see each other within the measures, and you still had a nice evening or afternoon, that you could see each other from a distance. But at that time at least it felt like I still had some social contact." – interviewee 13, male 25 years old*

Despite the large impact on well-being, Suffering-Followers continued to comply with the measure. Suffering-Rulebreakers, on the other hand, deviated more and more from the measure for the sake of their well-being; by deviating and making the rule 'fit', they felt better.

For many interviewees, in all groups, the VRP of one did not make sense anymore and was ‘practically inconvenient’. In many cases visitors come in pairs, for example if you want to invite a couple, your parents, your sister and her partner etc. Interviewees asked themselves why they were only allowed to invite one visitor and not one household, as according to them the risk of getting infected with COVID-19 would be the same. In practice, for Rulebreakers this resulted in relaxing the measure to allow one household instead of one person.

*"It could happen though that the two of us are somewhere, so like yesterday I was with my girlfriend at another friend's house. Yeah, that's not actually allowed. On the other hand, I think if I have COVID-19, my girlfriend probably has it too." – interviewee 8, male 17 years old*

Resilient-Followers looked at the measure level-headed, but found it practically inconvenient (e.g., if they wanted to invite a couple), but this did not lead to rule-breaking. One interviewee explained:

*"Both family and friends understand why we do it, so we don't evaluate it in terms of terrible and annoying, because that's not helpful. It doesn't make any sense to catastrophize it. You only make yourself unhappy with that. Of course, it was difficult to invite a limited number of people, but the people I know are rational and it didn't cause any problems. It's difficult to invite just one person when you invite a married couple. Then it’s hard to say, ‘you are allowed, and you are not’, so there are some people we haven't invited at this point that we normally would have invited.” – interviewee 2, male, 71 years old*

### Spring/summer (April-August 2021) – relaxation of VRP (max. two visitors, no restrictions) (*N* = 14)

For the Suffering groups, relaxation of the VRP from one to two visitors immediately led to improved well-being. Also, among Resilient-Followers people were happy with this small relaxation of the measure, but they were also levelheaded about it.

*"Relaxation, especially when you can invite two people again, is nice. But it's not the main thing for me, because life doesn't stop when there are restrictions." – interviewee 2, male 71 years old*

In the summer of 2021, there was a period without VRP. Interviewees in all categories took advantage of the relaxation, except for two Resilient-Followers for whom, due to a small social circle, it didn’t change much. Some of the Suffering groups met with large friend groups again. It was remarkable that several Followers indicated they did meet more friends, but they still received limited visitors at home. Risk perception due to, for example, vulnerable health or work played a role in this.

*"In two weeks, our middle one will have his birthday, and then we will just have another birthday party with time blocks to not exceed the number of people. We set the number at eight for this birthday, that's the maximum. I keep in mind a maximum number of people. Besides, we haven't started seeing people more often because the rule has been relaxed more." – interviewee 15, male 50 years old*

For several interviewees, being vaccinated – which might be a new ‘factor’ in making considerations in spring/summer 2021 – was not a reason to stop being cautious. Particularly within Resilient-Followers, interviewees took someone's vaccination status into account when meeting; some preferred to meet only with people who have been vaccinated.

*"If people let us know that they don't want to be vaccinated for whatever reason, then I don't make an appointment with those people. So yeah, that's too bad, but it’s not going to happen. I'm not taking any risks. Not for my own health, but also not for the people around me." – interviewee 10, male 65 years old*

This is also the case for Resilient-Rulebreakers, who assessed the risk of the situation and people they met.

*"We do make different considerations now. Either people have had a COVID-19 infection or people have been vaccinated, and then the risk feels lower to me. But four unvaccinated, COVID-19-naive people together, we wouldn't do that. So, we think about our choices and the consequences they could have. When we invite people, we think about who has which serology, who is vaccinated, who might have COVID-19." – interviewee 7, female 30 years old*

### Fall (November 2021) – increased stringency of VRP (max. four visitors) (*N* = 15)

After a summer with no VRP, the measure was introduced again in the fall of 2021, stating a maximum of four visitors. Resilient-Followers again had no difficulty in complying with the measure and it did not affect their well-being. They accepted the situation and again complied because of risk perception. Additionally, interviewees rarely met with more than four people, which made compliance easier. This also applied to Resilient-Rulebreakers. The pandemic changed formerly busy social schedules, which were now preferably quieter.

*"I don't notice it that much I guess, because I don't invite more than four people that often. That has changed since the pandemic and especially because you really speak to each other better and you have more in-depth conversations than just ‘how are you’. So, I think it's also genuinely more sociable and enjoyable." – interviewee 7, female, 30 years old*

Interviewees indicated that if the restriction changes to less than four visitors, there was a chance they would no longer comply to the measure, as they did earlier. Almost all Suffering-Followers complied with the measure again. Some had no problems with it, because they rarely invited more than four people at a time.

*"No problem, it's rare that we receive more than two guests. In this household it doesn't happen that often, so it doesn’t make any difference for us." – interviewee 10, male, 65 years old*

For others, the measure did affect well-being, but this was not a reason for non-compliance because of risk perception, a wish to follow the government rules and working in healthcare. For a few Suffering-Followers their ‘compliance limit’ was reached when the measure was reintroduced. Although they managed to comply throughout the pandemic despite the significant effect on well-being, now the measure was no longer followed. The interviewees did not completely disregard the measure, but they made different considerations than before. One interviewee mentioned that he invited just a little more people than the rule prescribed, because he and the people around him have been vaccinated and did a self-test beforehand.

*"In terms of group size, I was much stricter when no one had been vaccinated yet, or when only I had been vaccinated. You notice by being vaccinated you are more flexible. But I am strict about self-testing. If we consider inviting more people, then I really want everyone to do a self-test beforehand, just to protect each other." – interviewee 13, male, 25 years old*

The other Suffering-Follower interviewee indicated she struggled so much with having this measure implemented for the umpteenth time, that the VRP no longer determined her decisions. She looked at what number of visitors felt right.

*"I do think the most regrettable thing is that visitation is restricted again. I also notice friends don’t comply with it, even family members who were very strict at first. Today is my sister's birthday, which she celebrates with my parents for dinner. We agreed with the family that it would be okay if there are ten of us, if nobody has any symptoms and if we do a self-test beforehand. I notice that more and more people, like my friends, and even my strict family, look at how the measure can be circumvented. So now it's more about, at least around me, what people are comfortable with and not necessarily what the measure prescribes. Of course, what feels comfortable can be influenced by the measures, speaking for myself, but I notice it's not leading anymore." – interviewee 14, female, 25 years old*

Suffering-Rulebreakers indicated they experienced little inconvenience when the measure was introduced again. For them, it did not change much, mostly because they did not strictly comply with the measure anyway. Some of them still complied with the measure 'a little'. For example, one interviewee indicated that he applied for a maximum of ten visitors, because that did not feel like a major restriction and because it allowed him to see his group of friends. He considered social contact as more important than compliance, because it was necessary for his mental well-being.

*"I’m quite willing to invite fewer people, but whether it's four or eight... But I wouldn't have more than ten people at home. I want to adhere to it a little, but I don't feel like complying with all the rules very strictly now. Because I'm done with it and I think that the value of being able to have certain forms of social contact, that's worth more than the risk of getting COVID-19 from that." – interviewee 8, male, 17 years old*

## Discussion

The goal of this descriptive qualitative study was to examine the impact of the visitor restriction policy on compliance and well-being by following a cohort of 15 interviewees during the COVID-19 pandemic. The VRP appeared to be a measure with substantial impact on well-being for some and showed fluctuating compliance, in which feasibility and frequent changes in the VRP played a role.

When looking at well-being (Suffering/Resilient) and compliance (Followers/Rulebreakers), four different categories of people could be identified, who coped with the VRP in different ways and for different reasons. Both Following groups complied mainly because of risk perception and cue to action from the government, which seem to correspond with predictors of behavior from the Health Belief Model: perceived severity, susceptibility and cue to action [18]. The main difference between Resilient-Followers and Suffering-Followers was that the Resilient group accepted the situation and was not much affected in their well-being, while the Suffering group experienced a negative impact. Rulebreakers, on the other hand, did not always comply with the VRP. Both Rulebreaking groups made their own risk assessment, deviated when the measure was not considered logical to them and if the rule did not fit into their social structures. The main difference between Suffering-Rulebreakers and Resilient-Rulebreakers was that Suffering-Rulebreakers mainly deviated from the measure because they tried to find a balance between compliance and their well-being, while Resilient-Rulebreakers did not experience an impact on well-being.

A main finding of this study is that for most interviewees, especially among the Suffering groups, the VRP was perceived as an impactful measure, since it disrupted social structures. This is in line with previously mentioned studies, stating that social distancing rules require large behavior changes and are experienced as a high-cost measure [9, 14]. To miss out on important family meetings, support and closeness from friends and family had a negative effect on well-being. Suffering-Rulebreakers felt not seeing friends had a larger impact than getting infected would having); rule-breaking gave mental relief. Maintaining mental health and well-being as a reason for non-adherence is also found in literature [19]. Additionally, for some, non-adherence seems caused by the need to take control over their lives [10]. As seen among Suffering-Rulebreakers, they deviate to take control over their social needs and to keep themselves mentally healthy.

Another finding is that well-being seemed closely related to the number of visitors that was allowed, which showed a clear breaking point for all interviewees, including the most compliant ones. One visitor per day made it difficult to maintain existing social structures and felt not logical for most interviewees. Interviewees indicated that well-being improved when more visitors were allowed; four visitors was considered practically feasible, since most interviewees were used to inviting a maximum of four visitors, also before the COVID-19 pandemic. When less visitors were allowed, most interviewees showed a decrease in compliance and/or well-being, except for Resilient-Followers, for whom the number of visitors hardly affected well-being and compliance.

It also appeared that the frequent changes in the VRP negatively impacted well-being, future perspective and resilience of the Suffering groups. They were constantly challenged to change their plans when the VRP changed. While Suffering-Followers remained compliant, this was a reason for Suffering-Rulebreakers to deviate from the rule. This is in line with another study that found when policy decisions were experienced as too changeable, adherence might be affected [10]. This is, according to Williams et al. (2021) also the case for inconsistent and confusing rules, which we also found as a reason for non-compliance in both Rulebreaking groups. Only Resilient-Followers easily adjusted to the fluctuating pattern of the VRP, where acceptation of the situation seemed to play a role.

We took then background factors, age, gender, educational level, urban or rural living environment, health situation and current living situation of the participants into account when selecting the participants. However, we couldn’t see clear differences in our data when looking at background factors. Due to the small sample, examining the impact of demographic factors is difficult.

Although research [11] showed that youngsters did not always adhere to the rules, possibly because of their lower risk perception, in our study we see that the youngsters adhered to the rules. They had responsible jobs, or didn’t want to be the reason their grandparents got infected. Therefore, our data does not support this general observation. The youngsters in our research met more people then allowed because of their social structures. And when they met more people then allowed, the risks were calculated. The small sample could also be the reason we couldn’t conclude that being young and therefore belonging to a low risk-group was a reason for non-adherence to the VRP. Lastly, the four categories were quite stable during the various phases of the pandemic, meaning overall, interviewees continued to behave in a similar way, for similar reasons. Only a few interviewees ‘switched’ to another category (i.e., Suffering-Followers to Suffering-Rulebreakers) in the autumn of 2021 (the last period of stricter VRP). For some interviewees well-being was a decisive factor in the decision to stop complying. The long duration of the pandemic and repeated introduction of the VRP made that they could no longer cope. For some, vaccination played a role. It made them feel safe, which resulted in a lower risk perception and, therefore, different considerations regarding compliance. Also in other groups we observed new factors over time like vaccination (e.g., only inviting people who have been vaccinated or feeling more safe) and self-tests that influenced behavior. However, for some, vaccination was no reason to be less careful.

### Implications

Firstly, this study showed that the stringency of the VRP has a clear limit when it comes to well-being and compliance. During the winter of 2020–2021, when the VRP allowed one visitor, many interviewees showed a mental breaking-point. The restriction of one visitor had a great impact on well-being and showed consequences for compliance and support for the policy. For example, many interviewees mentioned that the policy was not logical anymore. They considered one visitor as risky as multiple visitors, when they are part of the same household. Thus, when it is difficult to understand why a certain VRP is implemented, support might be lower, possibly resulting in lower compliance. During future pandemics, it is therefore important to explain why certain choices are made. Additionally, policymakers should keep in mind that the VRP should be practically feasible to prevent non-compliance. For example, many interviewees often invited two people at the same time (e.g., a couple), which made one visitor very impractical. Results showed that a VRP that allowed four visitors was practically feasible, making compliance easier, with a smaller impact on well-being. These findings may help policymakers to weigh up the impact and the feasibility of a VRP next to epidemiological factors.

Secondly, when considering a VRP during a pandemic, it should be noted that people apply several strategies for having social contact to maintain well-being, that might undermine its goal to reduce infections. For example, celebrating Christmas and birthdays in small groups on multiple days or in time blocks on the same day, choosing the largest house (to keep enough distance), taking self-tests and meeting only with people who are vaccinated.

### Strengths and limitations

This study has notable strengths. First, this study had a longitudinal design, following a cohort of 15 interviewees from May 2020 until December 2021. Therefore, the results provide an in-depth view of compliance with the VRP and well-being during the different phases of pandemic. Second, every interviewee was interviewed by the same interviewer. The interviewer was aware of the story of the interviewee and a relationship of trust was build. Lastly, during the interviews, the researchers’ interpretation of the interviewees’ behavior was reflected upon to check whether the interpretation of the data was correct. Also, the assigned category was verified in the last interview round by asking the interviewee if they recognized themselves in the description.

However, this study also has some limitations. Interviewees were selected from respondents of the large quantitative cohort study of the RIVM, GGD GHOR and 25 GGD’s, which was not representative of the Dutch population. The interviewees were mostly highly educated people with no migration background, who were generally compliant (e.g., rulebreakers deviated from the rule only when it was necessary to regain well-being and made deliberate risk assessments). As the sample of interviewees was not representative, other relevant experiences could be missed in this research.

The interviewees were interviewed about different measures taken by the government, besides the VRP. When analyzing the interviews in relation to VRP we observed a relation between the compliance with the VRP and other measures (e.g., 1,5 m distancing rule, closing of places where people usually meet). This association was beyond the scope of the current study. However, future studies might consider the dependence between different measures.

Lastly, the measures taken to prevent the spread of COVID-19 started at March 2020 in the Netherlands. It took time to set up the study and the data collection started on May 1st 2020.Therefore we miss the information about the very beginning of the pandemic and the start of the restrictions. However, we assume that starting after several weeks after the official start of the pandemic didn’t affect our analysis much. Funding ended in December 2021, therefore the interviews stopped while the restrictions were still applied.

## Conclusion

With this longitudinal qualitative study, we aimed to observe the impact of the VRP on compliance and well-being by following a cohort of 15 interviewees during 12 interview rounds over a period of 21 months during the COVID-19 pandemic (May 2020-December 2021). The VRP appeared to be a measure with substantial impact on well-being for some and showed fluctuating compliance, in which feasibility and frequent changes in the VRP played a role. This study showed that four categories can be identified when observing the impact of the VRP on well-being and compliance. Some follow the VRP, while others deviate, and some experience a lower well-being, while others are resilient. Well-being seemed related to the number of visitors that was allowed: four visitors was feasible, while one visitor resulted in a negative breaking-point in resilience, which had an impact on compliance even among the most compliant. Taken together, this study provides valuable insights into the implications of and compliance to a VRP during different phases of the COVID-19 pandemic, which may contribute to policymaking during future pandemics.

### Supplementary Information

**Additional file 1: Appendix 1. **Overview of interview rounds and VRP per interview round.

## Data Availability

The datasets (recordings and transcripts) generated and/or analyzed during the current study are not publicly available due to privacy reasons, but are available from the first author on reasonable request.

## Abbreviation

VRP: Visitor Restriction Policy. The Visitor Restriction Policy is one of the preventive measures that impacted social life during the pandemic, i. e. governmental advice to limit the number of visitors allowed at home per day
