# Supplementary material for: Crossroads of well-being and compliance: a qualitative cohort study of visitor restriction policy during the COVID-19 pandemic, the Netherlands, May 2020-December 2021
Source: BMC Public Health. 2024 Jan 13;24:175. doi: 10.1186/s12889-024-17665-0 (PMC10787453; doi:10.1186/s12889-024-17665-0)
Supplement: Supplementary file 1 — Additional file 1: Appendix 1. Overview of interview rounds and VRP per interview round. [file 12889_2024_17665_MOESM1_ESM.docx]

**Appendix 1**

*Overview of interview rounds and VRP^[[1]](#footnote-1)^ per interview round.*

| **Round of interview** | **VRP** | |
| --- | --- | --- |
| Round 1**:** 01-06 May 2020  (15 interviews in total) | 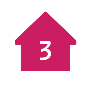 | At home up to three visitors are allowed |
| Round 2: 18-22 May 2020  (15 interviews in total) | 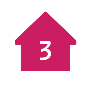 | At home up to three visitors are allowed |
| Round 3: 04-09 June 2020  (15 interviews in total) | 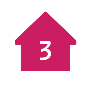 | At home up to three visitors are allowed |
| Round 4: 23-26 June 2020  (15 interviews in total) | 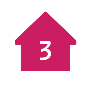 | At home up to three visitors are allowed |
| Round 5: 13-17 July 2020  (15 interviews in total) | 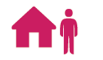 | There is no limit to the number of visitors |
| Round 6: 7-11 Sept 2020  (15 interviews in total) | 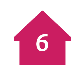 | At home up to six visitors are allowed (13 years or older) |
| Round 7: 19-23 Oct 2020  (15 interviews in total) | 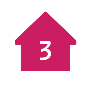 | At home up to three visitors are allowed (13 years or older) |
| Round 8: 27 Nov-3 Dec 2020  (15 interviews in total) | 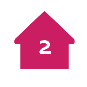 | At home a maximum of one visitor per day is allowed (13 years or older) |
| Round 9: 1-4 Feb 2021  (13 interviews in total) | 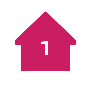 | At home a maximum of one visitor per day is allowed (13 years or older) |
| Round 10: 26-30 April 2021  (14 interviews in total) | 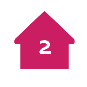 | At home a maximum of two visitors per day is allowed (13 years or older). People can receive maximum one visit per day. |
| Round 11: 24-31 Aug 2021  (14 interviews in total) | 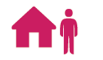 | There is no limit to the number of visitors |
| Round 12: 22-26 Nov 2021  (15 interviews in total) | 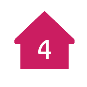 | At home a maximum of four visitors per day is allowed (13 years or older) |

1. Note: other measures, like keeping 1,5 meters distance, were also in force during the interview rounds. For example, people always had to keep a distance when visiting others or inviting others. [↑](#footnote-ref-1)
